# Using mechanism-based combinations of H_2_S-donors to maximize the cardioprotective action of H_2_S

**DOI:** 10.1007/s00210-023-02729-6

**Published:** 2023-09-29

**Authors:** Stella Ravani, Athanasia Chatzianastasiou, Andreas Papapetropoulos

**Affiliations:** 1 Center of Clinical, Experimental Surgery & Translational Research, Biomedical Research Foundation of the Academy of Athens, Athens, Greece; 2 Laboratory of Pharmacology, Faculty of Pharmacy, National and Kapodistrian University of Athens, Athens, Greece

**Keywords:** Hydrogen sulfide, Ischemia–reperfusion, Infarct, Heart, Cardioprotection, Nitric oxide

## Abstract

H_2_S-donors are cardioprotective in ischemia/reperfusion (I/R) injury. Some H_2_S-donors exert their beneficial effects in a nitric oxide (NO)-dependent manner, while others act using NO-independent pathways. The aims of the present study were to (i) evaluate whether H_2_S-donors with distinct pharmacodynamic properties act synergistically in I/R injury and (ii) determine if H_2_S-donors remain cardioprotective in obese mice. C57BL/6 mice were subjected to 30 min of ischemia followed by 120 min of reperfusion. Donors were administered intravenously at the end of ischemia (Na_2_S: 1 μmol/kg, GYY4137: 25 μmol/kg, AP39: 0,25 μmol/kg), while the 3-mercaptopyruvate sulfurtransferase (10 mg/kg) inhibitor was given intraperitonially 1 h prior to ischemia. Infarct size was estimated by 2,3,5-triphenyltetrazolium staining, while the area at risk was calculated using Evans blue. All three donors reduced infarct size when administered as a sole treatment. Co-administration of Na_2_S/GYY4137, as well as Na_2_S/AP39 reduced further the I/R injury, beyond what was observed with each individual donor. Since inhibition of the H_2_S-producing enzyme 3-mercaptopyruvate sulfurtransferase is known to reduce infarct size, we co-administered C3 with Na_2_S to determine possible additive effects between the two agents. In this case, combination of C3 with Na_2_S did not yield superior results compared to the individual treatments. Similarly, to what was observed in healthy mice, administration of a H_2_S-donor (Na_2_S or AP39) reduced I/R injury in mice rendered obese by consumption of a high fat diet. We conclude that combining a NO-dependent with a NO-independent H_2_S-donor leads to enhanced cardioprotection and that H_2_S-donors remain effective in obese animals.

## Introduction

Acute myocardial infarction (AMI) is a leading cause of morbidity and mortality worldwide (Timmis et al. 2022; Vos et al. 2012). Although advances in the treatment of AMI have translated into a considerable decline in mortality rates, heart failure (HF) remains a common complication of AMI, with an estimated incidence varying from 10 to 40% (Weir et al. 2006). The injury that the myocardium undergoes during ΑΜΙ, is due to both the ischemia and the subsequent therapeutic reperfusion. Reperfusion although beneficial, can itself induce myocardial damage and death (Yellon and Hausenloy 2007). Ischemia/reperfusion (I/R) injury is a complex process involving a multitude of triggers, mediators and effectors, each contributing to the final myocardial injury (Hausenloy and Yellon 2013; Heusch 2015). Ongoing research aims at discovering pharmacologic agents that when administered immediately prior to or at reperfusion, will be able to reduce myocardial infarct size.

H_2_S is a colorless, water-soluble, flammable gas with a characteristic unpleasant "rotten egg" odor. Until the 90s, it was studied exclusively for its toxic properties, but is now recognized as an important endogenous mediator (Cirino et al. 2023; Kimura 2014). Although H_2_S can be produced non-enzymatically, it is mainly generated through the action of three enzymes; cystathionine γ-lyase, cystathionine β-synthase and 3-mercaptopyruvate sulfurtransferase (Cirino et al. 2023; Filipovic et al. 2018; Wallace and Wang 2015). In the cardiovascular system, many studies have proven that H_2_S exerts beneficial/protective effects reducing blood pressure, promoting vasodilation, attenuating atherosclerosis and limiting endothelial dysfunction (Bibli et al. 2019; Coletta et al. 2012; Kanagy et al. 2017; Mani et al. 2013; Wang 2023; Wang et al. 2015; Yang and Wang 2015). In addition, H_2_S exerts antioxidant effects and stimulates angiogenesis (Filipovic et al. 2018; Kolluru et al. 2023; Papapetropoulos et al. 2009). Elrod et al., were the first to report on the cardioprotective effects of H_2_S in vivo; in a mouse I/R injury model, H_2_S limited oxidative stress and reduced infarct size (Elrod et al. 2007; Zhang et al. 2007). Many studies from several laboratories using H_2_S donors have confirmed and extended the finding that exogenously supplied H_2_S ameliorates I/R injury and reduces infarct size in rodents, pigs and dogs (Karwi et al. 2017a).

Several disease states are characterized by reduced levels of endogenous H_2_S (Cirino et al. 2023). For example, low H_2_S levels have been documented in heart failure, hypertension, atherosclerosis, diabetes and obesity. To effectively deliver H_2_S and replenish H_2_S levels, a variety of donors have been synthesized in recent years (Levinn et al. 2020; Szabo and Papapetropoulos 2017; Wallace and Wang 2015; Xu et al. 2019; Zheng et al. 2015). These donors differ in their pharmacokinetic profiles, the mechanism and the rate by which they release H_2_S. One distinct group of H_2_S donors that is widely used in the literature includes the sodium salts, sodium sulfide (Na_2_S) and sodium hydrosulfide (NaHS). These agents do not actually release H_2_S, but dissociate spontaneously to yield S^−2^ or HS^−^ and produce H_2_S in a pH-dependent manner (Papapetropoulos et al. 2015). Their administration causes an initial rapid increase in H_2_S levels, which then decreases with an equally rapid speed (Kashfi and Olson 2013). A second distinct group of H_2_S donors includes slowly releasing donors that were designed to mimic the endogenous rate of release of H_2_S; GYY4137 (morpholin-4-ium 4-methoxyphenyl (morpholino) phosphinodithioate) is the first in class of this compounds (Li et al. 2008). In addition, AP39 (10-oxo-10-(4-(3-thioxo-3H-1,2-dithiol5yl)-phenoxy)decyl triphenylphosphonium bromide belongs to the mitochondrial-targeted class of donors; its triphenylphosphonium group allows it to gain access to the mitochondrial compartment, while its dithiolethione group releases H_2_S slowly (Szczesny et al. 2014).

Several mechanisms have been implicated in the cardioprotective actions of H_2_S donors, including SAFE, RISK and the NO/cGMP pathways, antioxidant and anti-inflammatory effects, as well as mitochondria-related processes (Donnarumma et al. 2017; Li et al. 2018). Our group has previously compared the cardioprotective mechanisms employed by the fast donor Na_2_S, the slowly releasing H_2_S donors GYY4137 and thiovaline, and the mitochondrial-targeted donor AP39. We observed that although all of the donors reduced infarct size to a similar extent, only the effect of Na_2_S was eNOS-dependent (Chatzianastasiou et al. 2016). Thiovaline, GYY4137 and AP39 acted independently of the NO/cGMP pathway.

The aim of the present study was to investigate whether co-administration of H_2_S donors with distinct mechanisms of action, Na_2_S, GYY4137 and AP39, shows additive effects, leading to an increased cardioprotective effect. As almost all studies so far with H_2_S donors in I/R injury have been done in healthy animals, we also tested the ability of H_2_S donors with different mechanisms of action to limit myocardial injury after ischemia/reperfusion in mice with comorbidities using obese mice.

## Materials and methods

### Reagents

Sodium GYY4137 and AP39 were synthesized as previously described (Le Trionnaire et al. 2014; Alexander et al. 2015). Compound 3 (2-[(4-hydroxy-6-methylpyrimidin-2-yl)sulfanyl]-1-(naphthalen-1-yl)ethan-1-one), abbreviated as C3 was purchased from MolPort (Riga, Latvia). Na_2_S, TTC (2,3,5- triphenyltetrazolium chloride), Evans Blue Dye (tetrasodium (6E,6'E)-6,6-[(3,3'-dimethylbiphenyl-4,4'-diyl)di(1E)hydrazin-2-yl-1-ylidene]bis(4-amino-5-oxo-5,6-dihydronaphthalene-1,3-disulfonate)) were purchased from Sigma-Aldrich Chemie GmbH (Taufkirchen, Germany). HFD (E15744-34) and CD (E157452) were purchased from Ssniff (ssniff-Spezialdiäten GmbH, Germany).

### Animals

All animals used for experiments were bred/housed in individual cages under specific pathogen-free, temperature controlled (20–25 °C) and 12-h light/dark cycle conditions in full compliance with the guidelines of the Federation of Laboratory Animal Science Association recommendations in the Laboratory Animal Unit of the Hospital “Evangelismos” and allowed free access to diets and water. All studies were performed on male 8- to 16-week-old C57BL/6 J mice which were purchased from Alexander Fleming Institute (Athens, Greece). Mice were randomly assigned to diet or intervention groups. All animal procedures were in compliance with the European Community guidelines for the use of experimental animals; experimental protocols and all experimental procedures reported here were approved by the veterinary authority of the Prefecture of Athens, in accordance with the national Registration (Presidential Decree 56/2013) in harmony with the European Directive 63/2010.

To induce obesity, mice were fed for 10 weeks with a high fat diet (HFD) containing 45% calories from fat, 20% calories from protein, and 35% calories from carbohydrates. Control diet (CD) containing 10% calories from fat, 20% calories from protein and 70% calories from carbohydrates was used as the control diet. In all other experimental procedures, mice were fed a normal-fat regular diet (4RF22; Mucedola).

### Ischemia–reperfusion injury model *in vivo*

Male mice (8–12 weeks old in the normal diet groups and 18–22 weeks old for the HFD/CD mice) were randomly divided into groups and anesthetized by intraperitoneal injection with a combination of ketamine and xylazine (0.01 mL/g, final concentrations of ketamine, xylazine 10 mg/mL and 2 mg/mL, respectively). Anesthetic depth was evaluated by the loss of pedal reflex to toe-pinch stimulus and breathing rate. A tracheotomy was performed for artificial respiration at 120–150 breaths/min and a volume of 0,2 mL. A thoracotomy was then performed between the fourth and fifth rib and the pericardium was carefully retracted to visualize the left anterior descending (LAD) coronary, which was ligated using a 6–0 silk suture (W888; Ethicon) placed 3 mm below the tip of the left atrium with the help of 5 mm piece of a 1 mm diameter catheter tube. The heart was allowed to stabilize for 20 min prior to ligation to induce ischemia. After the ischemic period, the ligature was released and allowed reperfusion of the myocardium. Throughout experiments, body temperature was maintained at 37 ± 0.5 °C by way of a heating pad and monitored via a thermocouple inserted rectally. After reperfusion hearts were rapidly excised from mice and directly cannulated and washed with normal saline for blood removal. Hearts were perfused with 400 μl 1% Evans blue, diluted in normal saline. Hearts were kept at -80 °C for 1 h and then sliced in 2 mm sections parallel to the atrioventricular groove. The slices were incubated in 2 mL of 1% TTC phosphate buffer (PBS pH = 7,4) at 37 °C for 15 min and then fixed in 10% formaldehyde overnight. Slices were then compressed between glass plates 1 mm apart and photographed with a Cannon Powershot A620 Digital Camera through a Zeiss 459300 microscope and measured with the NIH ImageJ. Measurements were performed in a blinded fashion. The myocardial area at risk as well as the infarcted and the total area were automatically transformed into volumes. Infarct and risk area volumes were expressed in cm^3^ and the percentage of infarct-to-risk area ratio (%I/AAR) and of area at risk to whole myocardial area (% AAR/All) were calculated.

### Experimental protocol

Wild-type (WT) C57BL/6 J male mice were subjected to 30 min regional ischemia of the myocardium followed by 2 h of reperfusion with the following interventions. Control group (n = 10); Na_2_S group (n = 8): Na_2_S was administered as an IV bolus dose of 1 μmol/kg at the 20^th^ min of ischemia; GYY-4137 group (n = 7): GYY-4137 was administered as an IV bolus dose of 25 μmol/kg at the 20^th^ min of ischemia; AP39 group (n = 9): AP39 was administered as an IV bolus dose of 250 nmol/kg at the 20^th^ min of ischemia; C3 group (n = 8): C3 was administered intraperitoneally at a dose of 10 mg/kg 1 h before ischemia; Na_2_S-AP39 (n = 8): AP39 was administered as an IV bolus dose of 250 nmol/kg at the 15^th^ min of ischemia and Na_2_S was administered as an IV bolus dose of 1 μmol/kg at the 20^th^ min of ischemia; Na_2_S-GYY-4137 (n = 7) group: GYY-4137 was administered as an IV bolus dose of 25 μmol/kg at the 15^th^ min of ischemia and Na_2_S was administered as an IV bolus dose of 1 μmol/kg at the 20^th^ min of ischemia; Na_2_S-C3 group (n = 7): C3 was administered intraperitoneally at a dose of 10 mg/kg 1 h before ischemia and Na_2_S was administered as an IV bolus dose of 1 μmol/kg at the 20^th^ min of ischemia; The doses used for each donor were chosen based on the literature, including previous work from our group (Chatzianastasiou et al. 2016; Szabó et al. 2011). Na_2_S and GYY-4137 were dissolved in water for injection (WPI), while AP39 and C3 were dissolved in dimethylsulfoxide (DMSO). For iv administration, the volume of injection was 50 μl and the final concentration of DMSO was 0.05%. We have previously established that DMSO at this concentration exerts no pharmacological effects on the outcome of I/R injury. For each of the donors, the dose used was the lowest dose that was maximally effective in reducing infarct size that did not affect blood pressure.

For the obesity study, mice were randomly assigned to HFD and CD groups. The weight of the experimental animals was recorded on a weekly basis. Mice were subjected to 30 min regional ischemia of the myocardium followed by 2 h of reperfusion with the following interventions. Control CD group (n = 6); Control HFD group (n = 6); AP39 CD group (n = 6): AP39 was administered as an IV bolus dose of 250 nmol/kg at the 20^th^ min of ischemia; AP39 HFD group (n = 6): AP39 was administered as an IV bolus dose of 250 nmol/kg at the 20^th^ min of ischemia; Na_2_S CD group (n = 7): Na_2_S was administered as an IV bolus dose of 1 μmol/kg at the 20^th^ min of ischemia; Na_2_S HFD group (n = 6): Na_2_S was administered as an IV bolus dose of 1 μmol/kg at the 20^th^ min of ischemia.

## Statistical analysis

Data are expressed as means ± SD. Student’s unpaired two- tailed t test was used for comparison between two groups, and one-way ANOVA was used to compare three or more groups followed by a post hoc test. Sample sizes are reported in the methods section and in the figure captions. P was considered significant when < 0.05. GraphPad Prism 9.0 software was used for statistical analysis.

## Results

### Na_2_S-GYY4137 co-administration offers additional protection against ischemia/re-perfusion injury

**Fig. 1:**
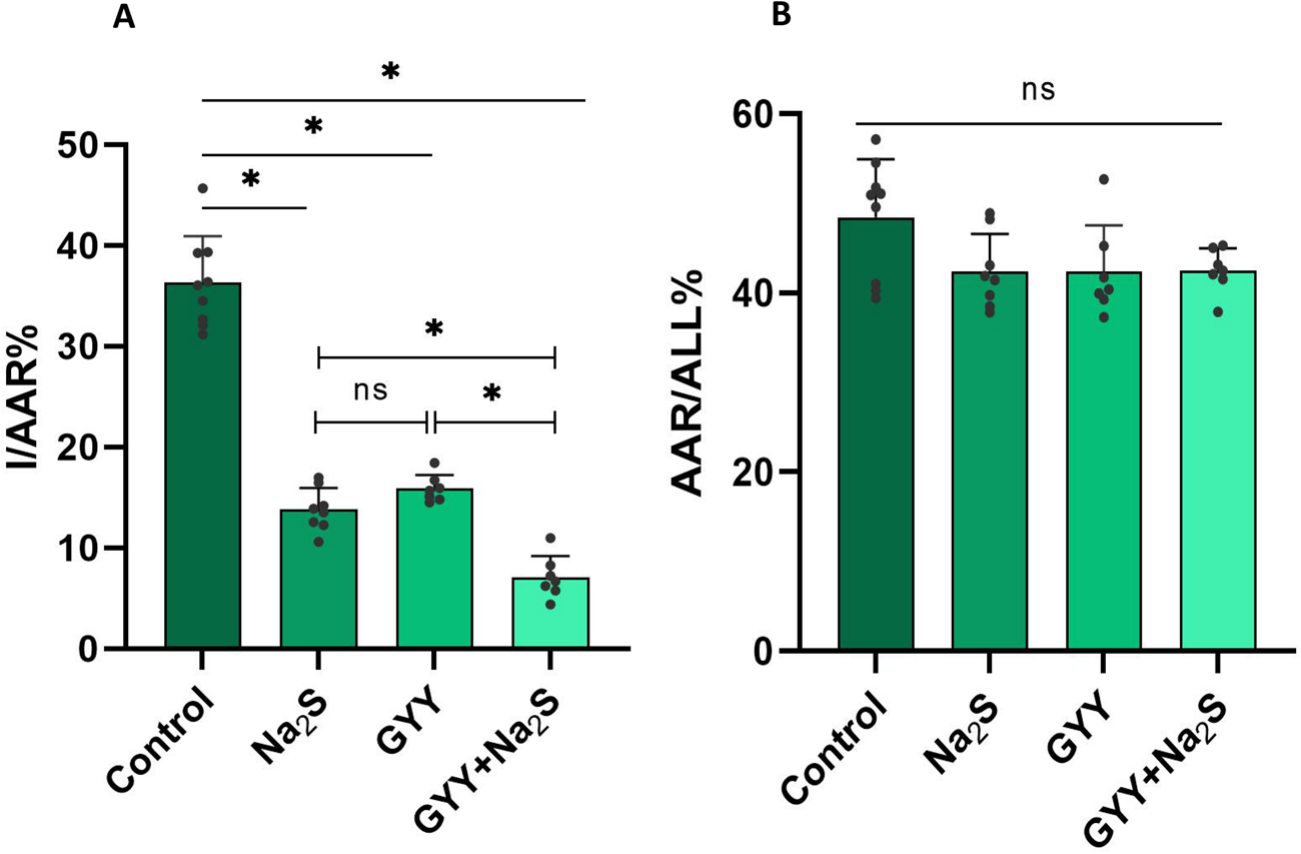
Na_2_S-GYY4137 coadministration reduces ischemia/reperfusion myocardial injury. **A** Comparison of the infarct area to area at risk ratio (I/AAR%) between Control, Na_2_S, GYY4137 and Na_2_S-GYY4137 groups. Mice were subjected to LAD ligation for 30 min; after removing the ligature, the hearts were reperfused for 2 h and stained as describe in the Methods section to determine the infarcted area and the area at risk. Na_2_S (1 μmol/kg) or GYY4137 (25 μmol/kg) were given intravenously at the 20^th^ minute of ischemia (10 min prior to reperfusion). For the combination treatment, GYY4137 was given at the 15.^th^ minute of ischemia followed by Na_2_S 5 min later. Data are presented as means ± SD; n = 7–9 mice/group; *p < 0.05 **(B)** The ratio area at risk/all of the Control, Na_2_S, GYY4137 and Na_2_S-GYY4137 groups was similar (ns p > 0.05)

Similarly to what has been described by us and others, administration of the H_2_S donor Na_2_S at the end of the ischemic period, caused a significant reduction in infarct size compared to the control group (Fig. 1A). GYY4137 that has been previously shown to exert a cardioprotective effect that is independent of ΝΟ, also attenuated the infarct size when administered at the end of the ischemic period. This reduction was to a degree that was similar to that observed with Na_2_S. When GYY4137 was combined with Na_2_S that exerts its infarct-reducing action in a NO-dependent manner, the observed effect was greater than that observed with Na_2_S or GYY4137 alone, confirming the existence of two distinct H_2_S-triggered cardioprotective pathways that can act in an additive fashion. In this series of experiments, the percentage ratios of area at risk to total area (AAR/All%) were also calculated to verify that all groups received the same degree of ischemic occlusion (Fig. 1B). No change in the AAR/All% was observed among groups.

### Co-administration of Na_2_S and AP39 reduces myocardial injury additively

**Fig. 2:**
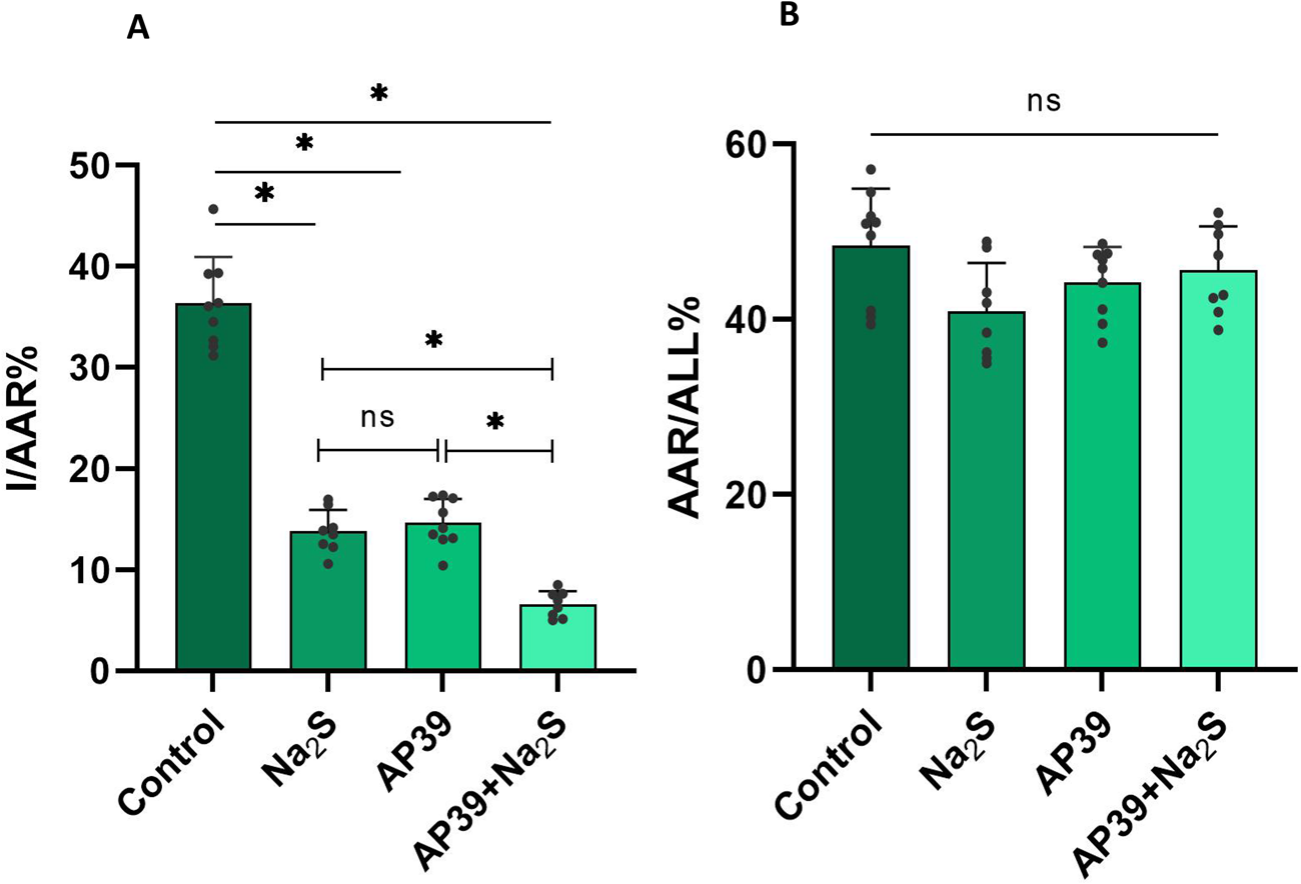
Na_2_S-AP39 coadministration diminishes infarct size. **A** Comparison of the infarct area to area at risk ratio (I/AAR%) between Control, Na_2_S, AP39 and Na_2_S-AP39 groups. Mice were subjected to LAD ligation for 30 min; after removing the ligature, the hearts were reperfused for 2 h and stained as describe in the Methods section to determine the infarcted area and the area at risk. Na_2_S (1 μmol/kg) or AP39 (250 nmol/kg) were given intravenously at the 20^th^ minute of ischemia (10 min prior to reperfusion). For the combination treatment, AP39 was given at the 15^th^ minute of ischemia followed by Na_2_S 5 min later. Data are presented as means ± SD; n = 8–9 mice/group; *p < 0.05 **(B)** The ratio area at risk/all of the Control, Na_2_S, AP39 and Na_2_S-AP39 groups was similar (ns p > 0.05). Mice in the control and the Na_2_S groups are the same as in Fig. 1

Infarct size was also reduced following administration of the mitochondrial-targeted H_2_S donor AP39. Interestingly, co-administration of Na_2_S and AP39, led to statistically significant cardioprotection over each individual donor. As shown in Fig. 2A, the percentage ratio of infarct to area at risk (I/AAR%) was 13.81 ± 2.13% for the Na_2_S group and 14.63 ± 2.39% for the AP39 group, while administration of both Na_2_S and AP39 resulted in enhanced cardioprotection (6.59 ± 1.29%). In this series of experiments, too, the AAR/All% were similar among groups (Fig. 2B).

### Combining a H_2_S donor with 3MST inhibition does not yield additional cardioprotection

**Fig. 3:**
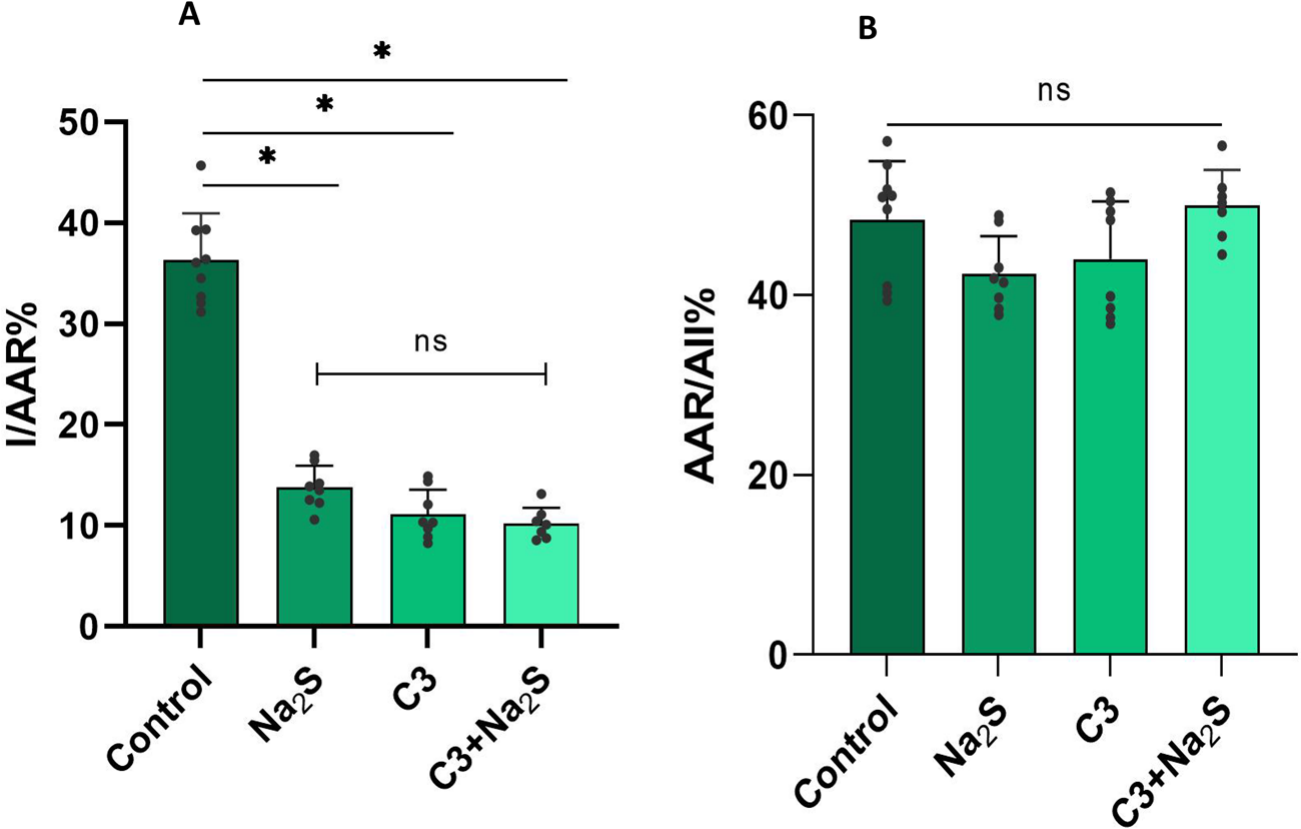
H_2_S donation (Na_2_S) combined with 3-MST inhibition (C3) has no additive effect on the reduction of infarct size. **A** Comparison of the infarct area to area at risk ratio (I/AAR%) between Control, Na_2_S, C3 and Na_2_S-C3 groups. Mice were subjected to LAD ligation for 30 min; after removing the ligature, the hearts were reperfused for 2 h and stained as describe in the methods section to determine the infarcted area and the area at risk. Na_2_S (1 μmol/kg, iv) or C3 (10 mg/kg, ip) were given as follows. Compound 3 (C3) was given 1 h prior to ischemia and Na_2_S was given at the 20^th^ minute of ischemia (10 min prior to reperfusion) both for the individual and the combination treatments. Data are presented as means ± SD; n = 7–9 mice/group; *p < 0.05 **(B)** The ratio area at risk/all of the Control, Na_2_S, C3 and Na_2_S-C3 was similar (ns p > 0.05). Mice in the control and the Na_2_S groups are the same as in Fig. 1

We have previously shown that mice lacking 3MST show reduced infarct size following ischemia/reperfusion injury and that pharmacological inhibition of 3MST also results in cardioprotection. In the present series of experiments, we observed that although Na_2_S and Compound 3 (C3) each reduced infarct size, coadministration of the two agents did not result in further inhibition of the ischemia/reperfusion injury (Fig. 3A). Our results are validated by the finding that the AAR/All% were not different in the groups tested (Fig. 3B).

### Cardioprotective effect of H_2_S donors in obese mice

Comorbidities can affect various cell signaling pathways and potentially eliminate the cardioprotective effect of a pharmacological agent (Ferdinandy et al. 2014). Obesity is a risk factor and can be characterized as a comorbidity in patients with AMI (Powell-Wiley et al. 2021).

**Fig. 4:**
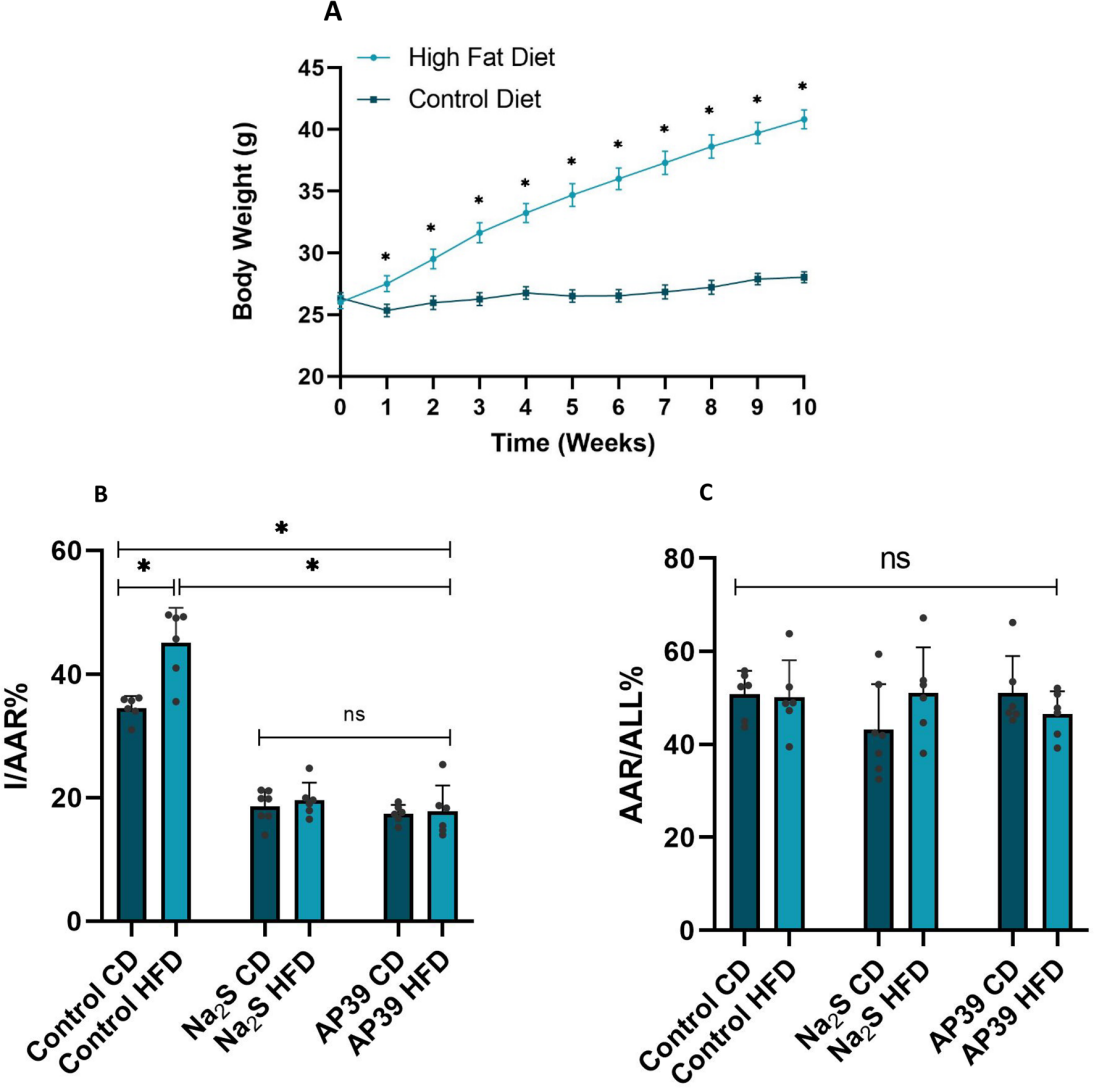
H_2_S donors, Na_2_S and AP39 are cardioprotective in obese mice. **A** 6-week-old male WT mice were fed a control diet (CD; 10% calories from fat) or a high fat diet (HFD; 45% calories from fat) for 10 weeks, and body mass was measured weekly. Data are presented as means ± SD; n = 21 mice/group; *p < 0.05 from control diet. **B** Comparison of the infarct area to area at risk ratio (I/AAR%) between Control, Na_2_S and AP39 in CD and HFD fed mice. Mice were subjected to LAD ligation for 30 min; after removing the ligature, the hearts were reperfused for 2 h and stained as describe in the methods section to determine the infarcted area and the area at risk. Na_2_S (1 μmol/kg) or AP39 (250 nmol/kg) were given intravenously at the 20.^th^ minute of ischemia (10 min prior to reperfusion). Data are presented as means ± SD; n = 6–7 mice/group; *p < 0.05. **(C)** No statistically significant difference of the ratio area at risk/all between Control CD/HFD, Na_2_S CD/HFD, AP39 CD/HFD groups (ns p > 0.05)

To test the efficacy of H_2_S donors in obese mice we used a diet-induced obesity model. As shown in Fig. 4A, the weight of the mice that received a CD increases only slightly over the 10-week period, with an average initial weight of 26.57 ± 2.02 g and an average weight after 10-weeks of 28.03 ± 2.00 g. On the other hand, the weight of the mice fed a HFD increased significantly over the 10-week period (mean starting weight 26.02 ± 2.46 g vs mean weight after 10 weeks 40.83 ± 3.58 g).

Obese mice suffered increased myocardial injury after ischemia/reperfusion compared to the control CD group (34.55 ± 1.94% vs 45.07 ± 5.70%; Fig. 4B). Administration of Na_2_S caused a significant reduction in infarct size compared to the control group in both CD and HFD groups. A reduction of similar magnitude was also observed when AP39 was given to obese and normal mice, suggesting that H_2_S donors retain their cardioprotective efficacy, irrespectively of their mechanism of action (NO-dependent vs NO-independent). The percentage ratios of AAR/All% was similar between the groups studied (Fig. 4C).

## Discussion

A variety of H_2_S donors have been tested and have shown efficacy in preclinical I/R injury models (Karwi et al. 2017a, b; Li et al. 2018; Szabo and Papapetropoulos 2017). In some cases, the donors were administered prior to the ischemic insult, a condition that is not clinically relevant. More often, the H_2_S donor was given at the end of the ischemic period or at reperfusion to mimic the real-life conditions encountered during medical care of cardiac patients. The donors used that have been proven to be effective in limiting infarct size in animal models include the fast releasing sulfide salts (sodium sulfide and sodium hydrosulfide) (Bibli et al. 2015; Chatzianastasiou et al. 2016; Elrod et al. 2007), the prototype slow-releasing H_2_S donor GYY4137 (Chatzianastasiou et al. 2016; Karwi et al. 2016), the mitochondrial targeted donor AP39 (Chatzianastasiou et al. 2016; Karwi et al. 2017a, b), the thioaminoacid thiovaline (Chatzianastasiou et al. 2016), the pH-activated H_2_S donor (Kang et al. 2016), esterase-activated prodrugs (Zheng et al. 2017), thiol-activated compounds (Kang et al. 2016), naturally occurring polysulfide donors (Bradley et al. 2016; Predmore et al. 2012), cysteine-persulfides (Griffiths et al. 2023) and hydro-persulfides (Pharoah et al. 2022). In most of the published studies, although efficacy was demonstrated, the molecular pathways through which the donor exerts its effects was not investigated. Preservation of mitochondrial function and anti-oxidant effects actions have been reported to underlie the acute effects of H_2_S donors on I/R injury (Donnarumma et al. 2017).

One pathway that has been shown to mediate many of the effects of H_2_S in the cardiovascular system is the NO/cGMP pathway (Donnarumma et al. 2017; Szabo 2017). Work by others and us, has shown that pharmacological or genetic inhibition of eNOS blocks the infarct reduction brought about by sulfide salts (Bibli et al. 2015; Chatzianastasiou et al. 2016; King et al. 2014). Unlike NaHS and Na_2_S, we observed that thiovaline, GYY4137 and AP39 inhibited infarct size in a NO-independent manner in mice (Chatzianastasiou et al. 2016). In partial agreement to our findings, Karwi et al. observed that GYY4137 limits infarct size in rats through the PI3K/Akt/GSK-3β pathway, as inhibition of PI3K resulted in complete reversal of the protective effect of GYY4137 (Karwi et al. 2016). However, eNOS inhibition in rats reduced, albeit marginally, the effect of GYY4137 on infarct size, suggesting that NO plays a minor role in GYY4137 cardioprotection in this species. Both our work and that of Karwi et al., confirmed the NO-independent effect of AP39 on infarct size in rats and mice (Chatzianastasiou et al. 2016; Karwi et al. 2017a, b). The cardioprotective effect of AP39 in I/R is also independent of cytosolic kinases. It results from inhibition of mPTP opening through a CypD-independent mechanism, as well as from reduction in mitochondrial ROS production (Chatzianastasiou et al. 2016; Karwi et al. 2017b). Herein, we tested whether using a combination of donors with distinct mechanisms of action (NO-dependent and NO-independent) would provide additive or synergistic effects. Indeed, we have found that co-administration of Na_2_S and GYY4137 or Na_2_S and AP39 provides greater cardioprotection than each of the donors when used individually. These results confirm that H_2_S donors utilizing different signaling pathways to exert their protective effects work additively and provide the rationale for using a combination of donors to secure maximal cardioprotection after I/R.

Endogenously produced H_2_S has been shown to be essential for cardiovascular homeostasis (Kolluru et al. 2023). Several studies have demonstrated that CSE-derived H_2_S is important in cardiac physiology and exerts protective actions against heart disease (Cirino et al. 2023; Polhemus and Lefer 2014). Mice overexpressing CSE in cardiomyocytes (Elrod et al. 2007) or in endothelial cells (Xia et al. 2020) exhibit smaller infarcts after I/R, while mice lacking CSE suffer more severe cardiac damage in I/R injury models compared to wild-type animals (King et al. 2014). Similarly, mice with targeted deletion of CSE have significantly greater cardiac enlargement, greater pulmonary edema and left ventricular cavity dilatation and exhibit exacerbated cardiac dysfunction in pressure overload-induced heart failure (Kondo et al. 2013). Unlike what has been observed with CSE knockout mice, mice lacking 3MST are protected from myocardial ischemia (Peleli et al. 2020). Since 3MST KO mice did not benefit from ischemic pre- conditioning, the smaller myocardial damage in 3MST KO was proposed to be due to increased oxidative stress present in 3MST KO hearts, that prepares the myocardium against the I/R injury. Contrary to the findings in the acute I/R injury model, mice lacking 3MST, like CSE KO mice, displayed exacerbated heart failure with reduced ejection fraction phenotype after pressure overload (Li et al. 2022). While trying to unravel the reasons for the differential responses of CSE KO to 3MST in heart disease models, it should be kept in mind that the localization of the two enzymes differs: CSE is present in the cytosol, while 3MST resides in the mitochondria. Based on our previous observations for the protective action of 3MST inhibition in I/R injury, we tested whether blockade of this enzyme combined with simultaneous H_2_S donation would yield additive or synergistic effects. Our observations that individual treatments reduced infarct size, but co-treatment did not lead to additive effects, indicated that this is not a useful drug combination.

Many of the failures in translating the efficacy of treatments with compounds that reduce infarct size in animal models to humans have been attributed to the fact that humans who suffer an AMI also present with comorbidities and risk factors (Ferdinandy et al. 2023). Other factors have also been proposed to explain the lack of translation of preclinical findings, including reproducibility and rigor of animal experimentation, species differences, as well as the fact that patients with co-morbidities who suffer an AMI are already on medication that modifies cardioprotection, masking the effects of additional agents given at reperfusion (Kleinbongard et al. 2020; Lecour et al. 2021; Lefer and Marbán, 2017).

We, thus, proceeded to evaluate if H_2_S donors retain their efficacy in reducing infarct size in animals with co-morbidities. To determine if H_2_S can reduce I/R injury in obese mice, we used both types of donors: one that exerts its effects in a NO-dependent and one that acts independently of NO. Interestingly, obese animals exhibited greater myocardial infarcts compared to control mice. It should be noted that conflicting results for the role of obesity in rodents have been reported, with some studies showing that obesity has a cardioprotective effect (Edland et al. 2016; Inserte et al. 2019; Salie et al. 2014), while others report no difference or increased myocardial injury after ischemia/reperfusion (Guedes et al. 2019; Liu et al. 2014). The discrepancies could be due to strain differences (Inserte et al. 2019) or differences in the diet.

Acute administration of Na_2_S or AP39 reduced infarct size to the same extent in mice with normal weight and obese mice, suggesting that H_2_S donors, are also effective in non-healthy mice. Similarly to what we observed with obese mice, acute administration of sulfide salts reduced infarct size in diabetic mice (Lambert et al. 2014). Our predication was that the NO-dependent H_2_S cardioprotective action of sulfide salts would be attenuated in obese mice, since these animals exhibit endothelial dysfunction, reduced eNOS activity/function and lower NO levels (Lundberg and Weitzberg 2022). Since this was not the case, we conclude that obese mice have enough functionality of their eNOS/NO pathway to mediate the NO-dependent effect of fast releasing H_2_S donors, or that under conditions of low NO, sulfide salts utilize alternative pathways to exert their cardioprotective effects. It is worth mentioning that long-term administration of H_2_S donors, also ameliorates cardiac damage in diabetic cardiomyopathy and obesity-induced cardiac dysfunction (Barr et al. 2015; Sun et al. 2021).

**Fig. 5:**
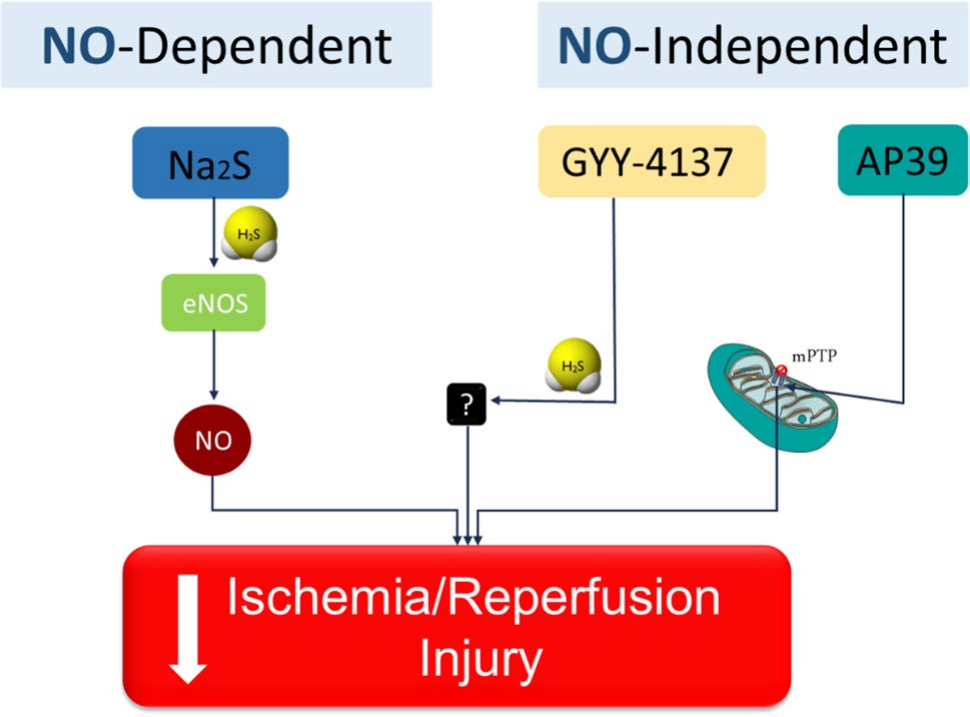
Schematic summarizing the pathways activated by NO-dependent and NO-independent H_2_S donors that lead to additive effects of the two classes of H_2_S donors

In conclusion, we provide evidence that combining H_2_S donors that use different mechanisms to protect the heart against I/R injury leads to an additive cardioprotective effect (Fig. 5). Moreover, H_2_S donors are effective in obese animals, providing the rationale for further pre-clinical testing in animals with other diseases associated with myocardial infarction and paving the way to test these agents in the clinic.

### Limitations of the study and future directions

In our experiments, infarct size and cardiac damage is assessed two hours post reperfusion, at a time point that is quite close to the onset of injury and rely on a single type of histochemical measurement. It would be useful to confirm our findings using a biomarker, such as troponin release and to evaluate the effectiveness of H_2_S donor treatment and their combination in leukocyte infiltration and inflammation in the heart, changes in cardiac metabolism, remodeling and fibrosis and to assess whether H_2_S donor treatment could prevent or delay the appearance of heart failure post myocardial infarction. Additionally, our study was conducted in male animals. Given the differences in drug responses that exist between males and females, it would be important to determine if our observations hold true in female mice. Useful insights might be gained by using combinations of submaximal concentrations of H_2_S donors to observe if additive or synergistic effects occur. Finally, it would be important to assess in more detail the underlaying molecular mechanisms that mediate the NO-dependent and NO-independent effects of H_2_S donors.

## Data Availability

Raw data from the experiments shown are available to referees and Editors of NSAP upon request.

## Abbreviations

3MST: 3-Mercaptopyruvate sulfurtransferase
AAR/All: Area at Risk/All
Akt: Protein Kinase B
AMI: Acute Myocardial Infarction
AP39: 10‐Oxo‐10‐(4‐(3‐thioxo‐3H‐1,2‐dithiol‐5yl)phenoxy)decyl) triphenylphosphonium bromide
C3: 2-[(4-Hydroxy-6-methylpyrimidin-2-yl)sulfanyl]-1-(naphthalen-1-yl)ethan-1-one
CD: Control Diet
cGMP: Cyclic guanosine monophosphate
CSE: Cystathionine γ-lyase
CypD: Cyclophilin D
eNOS: Endothelial Nitric Oxide Synthase
GSK-3β: Glycogen synthase kinase-3 beta
GYY4137: [Morpholin-4-ium 4 methoxyphenyl(morpholino) phosphinodithioate]
H2S: Hydrogen Sulfide
HF: Heart Failure
HFD: High Fat Diet
I/AAR: Infarct/Area at Risk
I/R: Ischemia/Reperfusion
IV: Intravenous
KO: Knockout
LAD: Left Anterior Descending
mPTP: Mitochondrial permeability transition pore
Na2S: Sodium Sulfide
NaHS: Sodium Hydrosulfide
NO: Nitric Oxide
PBS: Phosphate-buffered saline
PI3K: Phosphoinositide 3-kinase
RISK: Reperfusion Injury Salvage Kinase
ROS: Reactive Oxygen Species
SAFE: Survivor Activating Factor Enhancement
TTC: 2,3,5-Triphenyl-tetrazolium chloride
WT: Wild type
